# Polydimensional Structure and Psychosocial Functions of the Direct Address in TV Series

**DOI:** 10.3389/fpsyg.2021.662215

**Published:** 2021-07-06

**Authors:** Carlo Galimberti, Antonio Bova, Carmen Spanò, Ilaria Vergine

**Affiliations:** Department of Psychology, Catholic University of the Sacred Heart, Milan, Italy

**Keywords:** argumentation, communicative interactions, conversation, direct address, media studies, subjectivity, intersubjectivity, TV series

## Abstract

Traditionally, in media studies research, the direct address or aside, i.e., a construction in which a speaker communicates a message directly to the audience breaking the continuity of the narrative flow, has been investigated mainly for its dramaturgical function. The present study aims to consider the direct address as a research object of the social psychology of communication to increase our understanding of this technique by going beyond the analysis of its dramaturgical function. In particular, the direct address will be examined through an integrated approach based on argumentative and conversational tools to highlight its less known polydimensional structure, i.e., diegetic and extra-diegetic dimensions and their interactions, and psychosocial functions, i.e., connecting the characters among each other within the show as well as with the audience. This objective will be achieved by analyzing two different direct addresses from the American TV series House of Cards. The analysis showed that the direct address performs its dramaturgical function by impacting both diegetic and extradiegetic levels. In the first case, as considered in previous studies, these plans are activated in parallel, aiming to build what we have defined as the “strategic subjectivity” of the character who employs this technique. Instead, in the second case—which comprises two direct addresses produced by two different characters—this technique involves the creation of what we will call “platforms of intersubjectivity.” In this occurrence, the dramaturgical action establishes a “bridge” between the diegetic and extradiegetic plans that act synergistically. In conclusion, the present study shows how an integrated approach based on argumentative and conversational tools of analysis permits to enlarge the traditional media studies perspective, highlighting the less investigated polydimensional structure and analyzing the psychosocial functions of the direct address, here considered as a research object of the social psychology of communication examined in its diegetic and extra-diegetic dimensions. The integration of the pragma-dialectical approach to argumentation with the interlocutory logic theory has brought to light a new modality of use of the direct address that can be termed “intersubjective aside,” a type of aside that can be added to the three already known, i.e., aside ad spectatores, monological aside, and dialogical aside.

## Introduction

As observed by media researchers (e.g., Marriott, 2007; Gerbaz, 2008; Brown, 2012; Birke and Warhol, 2017), the direct address technique, i.e., a construction in which a speaker communicates a message directly to the audience, breaking the continuity of the narrative flow, is an ideal crossroad for the articulation of the diegetic and extra-diegetic dimensions of a media text. The diegetic dimension is internal to the text's narrative, while the extra-diegetic dimension reaches out to the audience in the context of fruition. Accordingly, the direct address can be described as a monolog that can only be heard by the extra-diegetic audience, while the characters standing right next to the speaker are totally unaware of his or her speech. In other words, the distribution of information between characters and viewers is strictly dependent on the privileged relationship between those characters who have access to this technique and the public.

Traditionally, in media studies research, the direct address has been investigated mainly for its dramaturgical function. The present study aims to consider the direct address as a research object of the social psychology of communication, increasing our understanding of the direct address by going beyond the only analysis of its dramaturgical function. The direct address will be examined by adopting an integrated approach based on argumentative (van Eemeren and Grootendorst, 1992; van Eemeren, 2010) and conversational (Trognon and Batt, 2010) tools to highlight its less known polydimensional structure, i.e., diegetic and extra-diegetic dimensions and their interactions, and psychosocial functions, i.e., connecting the characters among each other within the show as well as with the audience. This objective will be achieved by analyzing the direct addresses' use in the American TV series *House of Cards* (2013–2018). In particular, to highlight the direct address's polydimensional structure and psychosocial functions, we will analyze two different direct addresses. In the first case, as considered in previous studies, e.g., Klarer (2014), the direct address is addressed *prevalently* to the extra-diegetic dimension. In the second case, instead, the direct address is addressed *mainly* to the diegetic dimension.

A Netflix Original conceived and produced by Beau Willimon and David Fincher, *House of Cards* is an adaptation of the homonymous British TV show broadcast by the BBC in 1990 on the novel by Michael Dobbs. The series centers around the ambitious US congressman Francis (Frank) J. Underwood, played by Kevin Spacey, and his equally ambitious wife, Claire Underwood, played by Robin Wright. Frank and Claire Underwood's primary goal in life is to climb the ladder of power up to the top, regardless of what this process might entail. In the construction of its storylines, the direct address stands as the peculiar trait of *House of Cards*. Within this TV show, its use comes out “to be one of the most conspicuous narrative features of the unfolding episodes” (Klarer, 2014, p. 206), affecting the plot's development, the depiction of characters' profiles, and the audience mode of reception.

In order to present our study, the present paper is structured as follows. In its first part, we will discuss the reasons underlying the growing interest, within media studies, for the investigation of the use of the direct address. Afterward, the object of study and the analytical approach adopted for the analyses will be described, thus providing the methodological and conceptual framework on which the present study is based. In the last part of the paper, the results of the analyses of the two direct addresses considered for this study will be outlines and discussed, followed by a concluding section that summarizes the main findings and comments on their strengths as well as limitations.

## Analysis of the Direct Address in Media Studies

In his seminal work, Pfister (1991) analyzes the features of plays in their dramatic and theatrical dimensions, ranging from Greek tragedy and comedy to contemporary theater, with a particular focus on the plays of William Shakespeare. Throughout the body of his exploration, Pfister provides systematic definitions of narrative techniques employed in the construction of representations. Specifically, and of relevance to the study presented here, the author defines the theatrical aside that directly addresses the audience as “aside *ad spectatores*,” or “direct address.” This in contrast to, respectively, the “monological aside,” i.e., a remark that occurs in dialogue but is not meant to be heard by any of the speaker's interlocutors, and the “dialogical aside,” i.e., a remark that is addressed to a specific hearer, but is heard by nobody else present but the intended hearer.

Thus far, most of the studies on the direct address in media objects have focused on the dramaturgical function of the aside *ad spectatores*, showing how the main function of this technique, through which the continuity of the narrative flow is suddenly broken, is to provide the audience with more background knowledge about a certain situation. For instance, Mittell (2006) highlights how the direct address emerges as a peculiar tool for bolstering narrative complexity as a new form of narrative discursive techniques applied to entertainment television. The diegetic representation constitutes a part of the narration, which is, in fact, stratified since it incorporates and reveals the functioning of the narrative strategies. According to this media scholar, audiences take pleasure in the diegetic twists and the exceptional storytelling techniques needed to pull off such machinations. In this scenario, the educated spectator, who is accustomed to serialized stories with multi-faceted characters, can focus on the diegetic content and the elements that point out its formal construction and reproduction. In recent work, Klarer (2014) focused on the same research object of our study, i.e., the direct addresses the TV series *House of Cards*, aiming to examine the narrative's effects produced by this technique. The author identifies the primary function of the direct address in the so-called “metalepsis,” or narrative transgression: “What makes the aside, both in the theater and in film, equally intriguing is its metaleptic quality” (2014, p. 210). In particular, according to this author, *House of Cards'* direct address combines two narrative agencies that initially appear to speak in unison—that is, as long as the protagonist seems to be a reliable narrator—but later on become discordant, thereby producing a composite format with great narrative potential.

On the other hand, an array of studies shows how the use of the direct address in TV shows can have the function to disclose a character's plans and thoughts and, by doing so, strengthening the connection between the diegetic and extra-diegetic dimensions. For instance, Kinney (2019), focusing on the many independent films of the 1960s that feature black actors in moments of direct address, explains how through this technique it was possible to create reciprocity and alignment between the actor and the extra-diegetic audience. In the same vein, Woods (2019) points out how two British television comedies, *Chewing Gum* and *Fleabag*, sought to build close connections with their extra-diegetic viewers primarily through the employment of the direct address. In particular, the author shows how, in both comedies, the direct address's use intensifies the embrace of bodily affect and intimate access to interiority, drawing the extra-diegetic audiences to characters' singular perspective, and creating an intensely affective comic intimacy between the protagonists and the extra-diegetic spectatorship. By focusing on the use of the direct address in TV commercials, Hilmes (1985) argues that, like a show, which entails open complicity between spectator and object-seen, the texts, through the direct address, become intermixed and interactive, favoring the spectators' sensation of participation. In addition, the presence of the direct address in the narrative of the storylines elicits fans' engagement and involvement in the possible advancements of a fictional text, as indicated by Walton (1990).

In this section, we have tried to show how, traditionally, in media studies research, the direct address has been investigated mainly for its dramaturgical function. However, in our opinion, the evident dialogical nature of the direct address calls attention to the necessity of looking at the effects produced by the use of this technique as the result of interactional processes occurring on both the diegetic and the extra-diegetic dimensions. And this in order to consider in pragmatic terms its effects on the diegetic and extradiegetic levels and the possible interactions generated between these two levels. To fill this gap in the literature, this work intends to highlight its less explored polydimensional structure and psychosocial functions. In the next section of the paper, the object of study and the analytical approach adopted for the analyses of two different direct addresses from the TV series *House of Cards* will be described, thus providing the methodological and conceptual framework on which the present study is based.

## Methodology

### Object of Study

To highlight the direct address's polydimensional structure and psychosocial functions, we will analyze two different direct addresses from *House of Cards*. The first example is Frank Underwood's second direct address from the first episode of the first season of the show, and we have named it “Welcome to Washington.” In this first example, Frank Underwood's direct address is addressed to the extra-diegetic dimension that reaches out to the audience in the context of fruition. The second example is Claire's direct address from the penultimate episode of the sixth, and last, season of the show, and we have named it “I know you saw it too.” In the second case, the direct address is addressed to the diegetic dimension internal to the text's narrative. We have selected for the analysis the above-mentioned direct addresses because they allow us to clearly show the polydimensional structure and psychosocial functions generated by the use of the asides in a TV series, going beyond the so far produced analysis and categorization of its functions. The two sequences were fully transcribed, adopting conversation analysis conventions for transcribing vocal conduct in talk-in-interaction (Sacks et al., 1974; see Appendix). Two researchers revised all transcriptions until a high level of consent (agreement rate = 95%) has been reached.

### Analytical Approach

The analysis of the two direct addresses considered for this study is based on an integrated analytical approach articulating argumentative and conversational tools of analysis.

In a first phase, to reconstruct the structure of the two direct addresses from an argumentative perspective, in line with other scholars (Kuhn, 1991; van Eemeren and Grootendorst, 2004; Weigand, 2006; Rigotti and Greco Morasso, 2009; Bova, 2019), we will refer to the arguments advanced by a character through the use of a direct address with the scope to support, explain, justify, and defend a standpoint. In this endeavor, the analytical approach for identifying and reconstructing the arguments advanced by a character through the use of a direct address is the pragma-dialectical approach to argumentation (van Eemeren and Grootendorst, 1992; van Eemeren, 2010). According to this approach, the speakers choose the types of arguments that are useful to support their standpoint or weaken the interlocutor's standpoint (van Eemeren and Grootendorst, 1992, p. 138). Using this approach as a guide for the argumentative reconstruction aims to produce an analytic overview of all argumentative components of a discourse, which points are at issue, and which explicit and implicit arguments are advanced. We believe that this model fits particularly this study context because it provides specific criteria for identifying the speaker's standpoint within the direct address and the arguments put forth in support of it.

Subsequently, to reconstruct the two direct addresses' structure from a conversational perspective, we will refer to the interlocutory logic theory (Trognon and Batt, 2010). This approach is a “global theory of the cognitive-affective-social organization of talk-in-interaction” (Trognon and Batt, 2010, p. 19), aiming to describe interlocutory events formally and to build a grammar of the types of dialogue in which we engage and their felicity conditions. Based on the dialogical revision of the Speech Act (Searle and Vanderveken, 1985; Vanderveken, 1990), this approach (Trognon, 2002) is used to explore the mechanisms of the communicative action. More specifically, preparatory conditions and sincerity conditions—being two crucial logical components of the illocutionary force (Searle and Vanderveken, 1985, p. 16–19)—will be the focus of our analysis because of their relevance for meaning negotiation process.

The integration of these two above-mentioned approaches aims to reconstruct the polydimensional structure, i.e., diegetic and extra-diegetic dimensions and their interactions, and psychosocial functions of the characters' direct addresses, i.e., connecting the characters among each other within the show as well as with the audience. We consider the interlocutory logic theory to be the best theoretical frame for analyzing the illocutionary dimension of the direct address, and the illocutionary analysis the ideal “companion” to the pragma-dialectical approach to argumentation. The analysis of direct addresses from this double perspective will give us access to the relational, i.e., illocutionary, and strategic, i.e., argumentative, layers of its discursive plot. Therefore, we will develop sound assumptions on the multiple discursive dimensions of direct address and build a meaningful hypothesis on the psychosocial functions underlying them.

## Analysis

### Example 1. “Welcome to Washington”

Frank Underwood, who had successfully supported the USA president in his election campaign, is passed over as the next Secretary of State. Consequently, he organizes his strategy by putting all of his energy into achieving this goal. In this scene, Frank and Claire Underwood are attending the New Year's gala. Frank Underwood directs his gaze into the camera, thus indicating that this message is meant for an audience outside of the diegesis we are immersed in, explaining his less-than-stellar views on newly elected President Garrett Walker, Vice President of the United States Jim Matthews, and White House Chief of Staff Linda Vasquez. Thanks to Frank Underwood's help, they won the election, and now it's their turn to reward him for it. Frank Underwood is currently the House Majority Whip but is looking for a more prestigious position. Below, we included the full transcription of this sequence.

In this sequence, Frank Underwood's direct address, due to its structure and organization of content, provides the audience with all the main elements characterizing this dialogical technique, from the direct look into the camera to the creation of an alternative space-time frame within the narrative. Frank Underwood's direct gaze into the camera indicates that his monolog is not simply a self-addressed soliloquy, but a carefully crafted speech directed to the viewer outside of the diegetic dimension. Our analysis will initially focus on the direct address' argumentative dimension, and subsequently we will focus on its illocutionary dimension.

Frank Underwood's direct address's argumentative reconstruction shows how he wants to convince the extra-diegetic audience, i.e., the people watching the show, that he deserves a more prominent political role than the one he has now. We can describe Frank Underwood's standpoint as follows: “*I deserve a prominent political role.”* We have identified three different types of arguments advanced by Frank Underwood to convince this audience that he deserves a more prominent political role.

**Example 1:** Welcome to Washington (S01, E01). Participants: Frank Underwood (FU).

1.	(voices in the background)
2. FU:	Oh – President-elect Garrett Walker (he turns towards the camera,
3.	clapping and indicating the president elect). Do I like him? No.
4.	Do I believe in him? That's beside the point. (he says “yes” with
5.	the head) Any politician that gets 70 million votes has
6.	tapped into something larger than himself. Larger than even me, as
7.	much as I hate to admit it. (he begins to walk and indicates the
8.	president) And look at that winning smile those, trusting eyes. I
9.	latched onto him early on and made myself vital. (he stops in
10.	front of the camera) After 22 years in congress, (he nods) I can
11.	smell which way the wind is blowing. (0.1)
12.	Oh, – Jim Matthews (he turns himself and indicates Matthews with
13.	the hand), his right honorable 10. vice-president. – Former
14.	governor of Pennsylvania. He did his duty in delivering the
15.	keystone state, bless his heart, and now they're about to put him
16.	out to pasture, (he indicates him with the hand). But he looks
17.	happy enough, doesn't he? (he turns towards the vice-president and
18.	then, towards the camera) = (0.2) = For some, – it's simply the
19.	size of the chair, (he walks again and gets a glass of wine)
20.	(0.2) Huh. = (0.2) = Linda Vasquez (he indicates her with the
21.	hand), – Walker's chief of staff. – I got her hired. She's a
22.	woman, check, and a latina, check (he indicates her with the
23.	hand), but more important than that, – she's as tough as a two-
24.	dollar steak. Check, check, check.
25.	(he stops in front of the camera) (2.0)
26.	When it comes to the White House, you not only need the keys in
27.	your back pocket, – you need the gatekeeper (he indicates his
28.	back with the hand) (0.2) (he walks again) As for me, – I'm just a
29.	lowly house majority whip. – I keep things moving in a congress
30.	choked by pettiness and lassitude. My job is to clear the pipes
31.	and keep the sludge moving. – But I won't have to be a plumber
32.	much longer. I've done my time
33.	(he points the finger at the camera).
34.	I backed the right man (he turns towards the stage). (0.3)
35.	(he turns towards the camera) Give and take. (0.1)
36.	Welcome to Washington (he raises his glass and walks away).

Frank Underwood's first argument is based on the importance of being ambitious, and he immediately wants the audience to know that he is very much ambitious. After the President-elected Garrett Walker, he introduces Vice President Jim Matthews. Franks Underwood describes him as a politician with no power: (turns 14–17) “He did his duty in delivering the Keystone State, bless his heart. Now they're about to put him out to pasture. But he looks happy enough, doesn't he?”. According to Frank Underwood, Vice President Matthews looks happy even if he does not have any power, since (turns 18–19) “for some, it's simply the size of the chair.” Frank Underwood tells the audience that the vice-presidency is not enough to give him the power he wants. He does not look for a nice, big chair. He wants the presidency, the real power. We can describe Frank Underwood's first argument by the following sentence: “For some, it's simply the size of the chair.”

Frank Underwood's second argument is based on the importance of being an experienced politician. He starts his direct address by introducing the President-elected Garrett Walker and informing the audience that he supported him during the presidential elections. Frank Underwood says that he spent 22 years in Congress, and after all this time, he acquired a crucial political skill: (turns 10–11) “I can smell which way the wind is blowing.” It does not matter if he believes in President-elected Walker's qualities or not. According to Frank Underwood, what is important is that he supported the right man. We can describe his second argument by the following sentence: (turn 32) “I've done my time.”

Frank Underwood's third argument is based on the importance of controlling people and information in a place like the White House and a city like Washington D.C. After introducing the President-elected Garrett Walker and Vice President Jim Matthews, he introduces a third character, Linda Vasquez, the White House Chief of Staff. Because of her role within the White House, Frank Underwood describes Linda as the person who controls who goes through the President's office. Linda is the White House gatekeeper, and Frank Underwood tells the audience that he has control over the gatekeeper because he (turn 21) “got her hired.” We can describe his third argument by the following sentence: (turns 26–27) “When it comes to the White House, you not only need the keys in your back pocket, you need the gatekeeper.”

The analytical overview of the argumentative reconstruction of Frank Underwood's direct address's is summarized below:

**Table:** 

Issue: Does Frank Underwood deserve a prominent political role?	
Frank Underwood's standpoint:	Yes, I deserve a prominent political role.
Frank Underwood's arguments:	(a) For some, it's simply the size of the chair;
	(b) I've done my time;
	(c) When it comes to theWhite House, you notonly need the keys in yourpocket, you need theback gatekeeper.

Turning to the reconstruction of the structure of Frank Underwood's direct address from a conversational perspective, the principal aim of this second phase of analysis is to describe the illocutionary mechanisms on which Frank Underwood's direct address is based. In particular, the analysis will now focus on the illocutionary force of the statements presupposed by the three arguments advanced by Frank Underwood, as identified in the first phase of the analysis.

Based on the dialogical revision of the Speech Act Theory (Searle and Vanderveken, 1985), the three statements presupposed by the three arguments advanced by Frank Underwood constitute the preparatory conditions of what is explicitly affirmed by the three arguments and therefore of the standpoint itself. A preparatory condition is a state of affairs that must be presupposed by the speaker in employing a particular illocutionary force, e.g., promising, advising, warning, asserting, etc., and it is a necessary condition for the non-defective employment of that force. In a real conversation, “in the performance of a speech act, the speaker presupposes the satisfaction of all the preparatory conditions” (Searle and Vanderveken, 1985, p. 17).

In the first example we have analyzed, Frank Underwood, acting in a fictional conversation, needs to explicit the presuppositions useful to build the conversational context with the extra-diegetic audience, i.e., the people watching the show. By doing so, Frank Underwood produces some effects psychosocial in nature. He does so in a 2-fold manner. First, by sharing his secret goal, i.e., (turns 31–32) I won't have to be a plumber much longer, with the audience, Frank Underwood puts himself on the border that divides the diegetic from the extra-diegetic dimension, accomplishing a double task: (a) at the diegetic level, he contributes to building his subjectivity; (b) at the extra-diegetic level, he proposes to the audience the role of the addressee of his asides. Second, by using irony and sarcasm, he exploits Grice's maxim of quality and engages the audience in cooperative actions, establishing illocutionary relationships with strong relational fallouts with the extra-diegetic audience (Vanderveken, 1990, p. 72–75).

A further step in producing some effects psychosocial in nature is performed by Frank Underwood's last remark, (turn 36) “Welcome to Washington.” According to Searle and Vanderveken (1985, p. 216), “to welcome somebody is to receive him with hospitality, and thus welcoming might be defined as an expression of pleasure or good feeling about the presence or arrival of someone. Welcoming… is essentially hearer-directed.” We believe that Frank Underwood's last remark can be defined as the pragma-semiotic root of this sequence. In fact, by saying “Welcome,” Frank Underwood manifests his intention to attribute a dialogical role to the extra-diegetic audience because it becomes an essential partner for the validation of his future direct addresses, leading them on a path that goes from the extra- to the intra-diegetic level. Besides, Frank Underwood also attributes a relational role to the extra-diegetic audience. It becomes a real partner in the narrative process by virtue of a complementary path going from the intra- to the extra-diegetic dimension. In the light of these considerations, we could say that the extra-diegetic audience became a reality composed of “required guests.”

In conclusion, the analysis of Frank Underwood's direct address by adopting an integrated approach based on the pragma-dialectical approach to argumentation and the interlocutory logic theory has shown how, by advancing arguments in support of his standpoint, Frank Underwood gives rise to an effective intertwinement among the actions accomplished by his talk, the discursive world, and the roles he proposes to the audience. The analysis also revealed the central role of the character: he is at the center of the mediation between diegetic and extra-diegetic dimensions that, as we noted earlier, are traditionally distinct. It is precisely on this “centrality” that Frank Underwood builds his “strategic subjectivity,” deciding which elements of his identity should come into play. We use the term “strategic” because his actions, on the one hand, aim at building relationships with the other characters and commenting on the events; on the other hand, they guide the audience in understanding the fictional events and orient it toward modes of fruition that respond to the dramaturgical plan.

### Example 2. “I Know You Saw It Too”

In the second scene selected for analysis, Claire Underwood—who in the sixth season decides to return to her maiden name, Claire Hale—has a sit-down interview on a national TV program, during which she announces that she is pregnant with a little baby girl (turn 8). She also depicts the figure of her dead husband, Frank Underwood, as a horrible person, an unknowable con man (turns 22–23). Doug Stamper, Frank Underwood's closest old friend and collaborator, watches this interview with his arms folded. Claire Underwood, through a dialogic direct address (cf. Pfister, 1991) directed to Doug Stamper, within the same diegetic environment, utters, (turn 23) “Come and get me, Doug.” Doug Stamper's undying friendship for Frank Underwood puts him into a fit of rage. At this point, Doug Stamper gets up from his couch, goes into the washroom, and shaves his beard, dramatically cutting off his face. Then, through a direct address directed to the audience in the extra-diegetic context of fruition, he says, (turn 24) “She leaves me no choice.” Below, we included the full transcription of this sequence. Claire Underwood's direct address (turn 23) and Doug Stamper's direct address (turn 24) are written in bold:

**Table:** 

1.	Interviewer:	I tested you **=** and you passed**=** It's basically how I see it Madame President
2.	CU:	You're impertinent (0.2) That was clear from the start
3.	Interviewer:	No, I'm ambitious**=** which is something that I've always admired from afar about you
4.	CU:	Even when you were attacking me?
5.	Interviewer:	Forgive me **=** I become aggressive when I'm nervous, (0.1) it's always been that way. Can we start over? **=** Because I think I finally understand you and what you're trying to do for this country. I can help you get your message out
6.	CU:	Sure:
7.	Interviewer:	Okay, let's begin.
8.	CU:	First things first **=** Despite what the odds makers have decided, (0.4) I'm going to be a mother of a baby girl
9.	Interviewer:	Wow! Congratulations::!
10.	CU:	Thank you:
11.	Interviewer:	And have you picked out a name?
12.	CU:	Not yet, (0.1) I need to see her face first
13.	Interviewer:	What do you most wish the American people knew about you?
14.	CU:	That I like nothing more than to laugh
15.	Interviewer:	And what does the prospect of becoming a mother **=** while being the leader of the free world, feel like?
16.	CU:	Bittersweet. I didn't expect to deliver Francis' child as a widow, of course (0.2) But I have things to do, for my unborn child, for this unformed nation **=** I want to create a progressive and productive Hale legacy for the ages
17.	Interviewer:	Speaking of which: why did you return to your maiden name?
18.	CU:	Well, I think your question answered itself: (0.2) maiden name. The assumption behind that expression led me back to what I can only call “my name”
19.	Interviewer:	President Hale, why the delay in nominating a replacement for the vice-presidential vacancy?
20.	CU: Claire:	Two reasons. (0.1) One, constitutionally Congress needs to confirm the choice, and our current Congress is dirty. (0.2) I'll have to see what happens with the mid-terms. And two, (0.1) if I have learned anything from partnership, you have to choose someone who'd take a bullet for you **=** and Melanie I'd like to come clean on something
21.	Interviewer:	Please
22.	CU:	The truth is, (0.2) and it pains me to say this, but the truth is: (0.1) this country never got to know the real Francis Underwood **=** I'm not sure I ever really knew him myself.
23.		**I know you saw it too. He was impossible to know, like all con men.** (0.1) **He****played****us all. Come and get me, Doug**.
24.	DS:	**She leaves me no choic**

Like the analysis of Example 1, here, too, our analysis will initially focus on the direct address' argumentative dimension, and subsequently we will focus on its illocutionary dimension. In particular, our analysis will be centered on Claire Underwood's direct address directed to Doug Stamper, within the same diegetic environment. This example of aside differs from the previous one for a basic dynamic that emerges as extremely significant. All the previous direct addresses in this TV series were directed to the audience in the extra-diegetic dimension. Instead, in this scene, Clair Underwood's direct address is decidedly unexpected because it is directed to another character, Doug Stamper, within the same diegetic environment, i.e., within the fictional frame of the narrative, and not toward the audience in the extra-diegetic setting. This is the first and only time, in this TV series, that the direct address is employed in this way. The first time that a fictional character addresses another character by adopting a specific modality that pertains to a form of communication conceived for bridging two distinct realities, i.e., the diegetic and extra-diegetic dimensions of a media text.

Claire Underwood's direct address's argumentative reconstruction shows how she wants to convince another character, Doug Stamper that Frank Underwood, who was her husband and Doug Stamper's old friend, was betraying everyone, including him. We can describe Claire Underwood's standpoint as follows: “*Frank Underwood was cheating us all*.” We have identified two different types of arguments advanced by Claire Underwood to convince Doug Stamper that Frank Underwood was deceiving all those around him.

Claire Underwood's first argument is based on the fact that she knows that Doug read Frank Underwood's secret diary—a collection of thoughts and revelations as expressed in all the direct addresses uttered by Frank Underwood throughout the previous seasons of the series. Therefore, as she does, Doug Stamper is also aware of all the strategic plans and horrible actions carried out by Frank Underwood to reach his own goals. According to Claire Underwood, Doug Stamper cannot deny that Frank Underwood was cheating him too, because she knows that he read Frank Underwood's secret diary. Accordingly, we can describe Claire Underwood's first argument by using the following sentence: “I know you saw his secret diary too.”

Claire Underwood's second argument is based on the typical traits characterizing a “con man.” Nobody knew that Frank Underwood was cheating everybody because he was a con man and, “like all con men,” he was a man impossible to truly know. The fact that both his wife, i.e., Claire Underwood, and his old friend, i.e., Doug Stamper, did not know that Frank Underwood was cheating everybody can be easily explained by the fact that he was a con man, that is, a manipulative individual. Accordingly, we can describe her second argument by using her own words: “He was impossible to know, like all con men.”

The analytical overview of the argumentative reconstruction of Claire Underwood's direct address is summarized below:

**Table:** 

Issue: Was Frank Underwood cheating us all?	
Claire Underwood's standpoint:	Yes, Frank Underwood wascheating us all.
Claire Underwood's arguments:	(a) I know you saw his secretdiary too;
	(b) He was impossible to know, like all con men.

Turning to the reconstruction of the structure of Claire Underwood's direct address from a conversational perspective, the principal aim of this second phase of analysis is to describe the illocutionary mechanisms on which Claire Underwood's direct address and Doug's successive one are based. In particular, the analysis will now focus on the illocutionary force of 23 CU and 24 D, that is the statements effectively uttered by the authors of the two direct addresses.

By adopting a psychosocial stance, we will pay attention to the conversational context of the two direct addresses, that is, to the twenty-two previous speech turns that we will consider with regards to both the speech act's propositional contents and the illocutionary force that appear in them. The consideration of Claire's speech turns from the point of view of propositional contents reveals the emergence of two lines of discourse: one linked to contents of private nature (I have things to do, for my unborn child; the choice of the maiden name), the other of an institutional nature (Hale legacy; I have things to do… for this unformed Nation; replacement of the vice-president).

The convergence of the two lines of discourse (13. I: What do you most wish the American people knew about you? 14. CU: That I like nothing more than to laugh; 15. I: “And what does the prospect of becoming a mother = while being the leader of the free world, feel like?”; 16. CU: “Bittersweet … But I have things to do, for my unborn child, for this unformed nation”) generates Claire's action program that combines the dimension of the attention to desire (unborn child) with that of the exercise of power (unformed Nation). In the commissive with which 16. CU concludes her speech turn (“I want to create a progressive and productive Hale legacy for the ages”), the action program concentrates into a declaration of commitment that synthesizes, in narcissistic terms, the private side (i.e., the exaltation of desire) and the public side (i.e., the exercise of power) that have characterized the entire progression of the interview.

The “turning point” represented by the convergence of the two lines of content also plays a fundamental role for the analysis of the illocutionary dimension. In particular, we refer to the “revelation” of the truth (23. CU: “The truth is,…”) regarding the “real” nature of Frank Underwood. This revelation completes the cognitive environment's construction that frames the two direct addresses that conclude the sequence.

Let us now consider the first direct address, 23. CU, made up of four speech acts, three assertive and one directive.

(a1) “I know you saw it too”: assertive that expresses a belief.
(a2) “He was impossible to know, like all con men”: assertive that express a judgment.
(a3) “He played us all”: assertive that expresses a judgment.
(d1) “Come and get me, Doug”: directive expressed by an imperative but that, as we will point out shortly, also represents a desire (Condoravdi and Lauer, 2012, p. 39).

According to the illocutionary analysis suggested by Beversluis (1971, p. 347) in some cases “I know that p” can be considered in the same way as “I warn you that p.” As it is also well-known, thanks to the semantic analysis of the verb “to warn” (Searle and Vanderveken, 1985), “I warn you that P” from the illocutionary point of view has a double nature and can be considered as either directive or assertive “about the state of affairs represented by P.” “I can warn (…) you that such and such is the case or I can warn (…) you to do something. But the two uses are not independent. When I warn (…) you that something is the case I am normally warning you that it is the case with a view to getting you to do something about it” (p. 202–203). In our opinion, this is precisely the case represented by the direct address made by CU to Doug. If we consider (turn 23) “I know you saw it too” as equivalent to “I warn you that I know you saw it too,” we can see that the speech acts which are part of the same speech turn concretize both the assertive component (turn 23 “He was impossible to know, like all con men. He played us all”) and the directive component (turn 23 “Come and get me, Doug”), following the binary structure that, as we have seen in carrying out the analysis of the propositional component, characterizes the entire sequence.

To complete the analysis of 23. CU, we should note that this first direct address remains within the perimeter of the diegesis. In other words, we can say that this direct address represents an invitation to construct an intra-diegetic intersubjective context, in the sense that Claire addresses Doug by exiting the conversation with the interviewer while remaining within the narrative of the story. Before proceeding with the analysis of 24. DS, the second direct address of the sequence under consideration, we would like to clarify the meaning adopt for the concept of intersubjectivity. For us, intersubjectivity is the phenomenological bridge between the subject and the other(s). It refers to “the process that allows actors to create a shared world within which they can interact with a good level of inter-comprehension, that is of mutual intelligibility of their communicative intentions” (Galimberti and Spanò, 2017, p. 196). The pillars of such a world are four specific properties that comprise modalities of action and, at the same time, results of the actors' actions.

The first characteristic of a shared world is “the construction of the actors' subjectivities and their mutual recognition by actors themselves.” The second one is “the conjoint definition of rules regarding interaction management and the relationships between actors according to situations.” The third feature is “the definition of the objects involved in the interaction” and, lastly, the fourth one derives from “the combination of conversational and discursive rules that allows actors to talk about the objects.” “These four characteristics demonstrate that co-referring to a shared world is a signal of a back reference to the actors themselves, that is: when actors talk about objects present within their world, they also give information about themselves, leaving clues, signs of subjectivity that are being elaborated during interaction” (Galimberti and Spanò, 2017, p. 196).

If we consider the speech act contained in 24. DS (“She leaves me no choice”), we can see that Doug makes Claire's directive (turn 23 “Come and get me, Doug”) successful and satisfied (Vanderveken, 1990, p. 129–134). This entails two consequences: (a) Doug accepts to enter the “intersubjective intra-diegetic platform” that Claire has built for him; (b) Doug relaunches to the audience the accepted contents (i.e., Claire's project of power and desire) making explicit his being “caught” in this game and shaping them into a new “intersubjective platform,” this time of extra-diegetic nature.

In summary, then, we can schematize the results of our analysis as follows: Direct address 1 = diegetic dimension = creation of an intersubjective context, of an “intersubjective platform (between Claire and Doug) of intra-diegetic nature.” Direct address 2 = extra-diegetic dimension = Doug accepts Claire's proposal, enters the “intersubjective intra-diegetic platform,” confirms its contents and commits himself to their realization. By addressing the audience, he re-launches Claire's proposal to the viewers, making it the content of a new “intersubjective platform,” this time of extra-diegetic nature.

Concluding remarks: (a) desire and power are the elements that allow to “manage” the simulations and manipulations of a “con man”; (b) the two direct addresses open and close the circle between “inside” and “outside” the narration, establishing a new modality of use of the direct address that, in our opinion, can be termed “intersubjective aside,” a mode that can be added to the three known and already described at the beginning of this work, that is: aside *ad spectatores*, monological aside, and dialogical aside.

## Discussion

The direct address constitutes one of the most interesting dramaturgical techniques used in performative arts. Traditionally, in media studies research, we have seen that the direct address has been investigated mainly for its dramaturgical function (e.g., Hilmes, 1985; Mittell, 2006; Klarer, 2014; Kinney, 2019; Woods, 2019). By adopting an integrated approach based on the pragma-dialectical approach to argumentation (van Eemeren and Grootendorst, 1992; van Eemeren, 2010) and the interlocutory logic theory (Trognon and Batt, 2010), the present study considered the direct address as a research object of the social psychology of communication and aimed to highlight its less known polydimensional structure and psychosocial functions. In this endeavor, we analyzed two different direct addresses from the American TV series House of Cards. In the first case, the direct address is addressed to the extra-diegetic dimension that reaches out to the audience in the context of fruition (cf. aside *ad spectatores*, in Pfister, 1991). In the second case, instead, the direct address is addressed to the diegetic dimension internal to the text's narrative (cf. dialogical aside, in Pfister, 1991). At this juncture, it seems appropriate to take stock of some findings of our study.

In line with previous studies, the findings of the analysis of the two examples of direct addresses show that the direct address not only permits the connection between diegetic and extra-diegetic dimension, but it also has the function of empowering the diegetic dimension by modifying the text's narrative. Considering the effects of the direct address on the extra-diegetic dimension, we showed how its use permits disseminating information to the audience, contributing to creating a frame of expression only for the characters and the audience. Through the analysis of the first example, we have seen how Frank Underwood distances himself from the world depicted on the screen and moves toward the world on the other side, and his direct address exemplifies the constant blurring of diegetic and extra-diegetic dimensions (Tindale, 2015). In this case, the direct address is employed as a strategic maneuvering (van Eemeren and Houtlosser, 2002) by Frank Underwood to persuade the extra-diegetic audience that he deserves a more prominent political role than the one he has till that moment. The reasons supporting his argumentative message relied on his personality and not, instead, on his political ideas' virtues. It is all about his personality, his skills, and his strengths, while there is no mention of what he wants to realize for his country. It's all about his personality, not about politics. In addition, the findings of the analysis of the two examples of direct addresses also bring to light that when the direct address is addressed to the extra-diegetic dimension that reaches out to the audience in the context of fruition, it conveys the characters' subjectivities to the audience.

As far as the diegetic dimension is concerned, in line with previous studies (e.g., Mittell, 2006; Klarer, 2014), we lit up how the direct address has the function of empowering the diegetic dimension by modifying the text's narrative. In the second example analysis, we have seen that Claire Underwood's direct address differs from the previous one because it is directed to another character, Doug Stamper, within the same diegetic environment, i.e., within the fictional frame of the narrative, and not the extra-diegetic audience. In particular, in this case, the direct address is employed as a strategic maneuvering (van Eemeren and Houtlosser, 2002) by Claire Underwood to convince another character, Doug Stamper, that Frank Underwood, who was considered by Doug Stamper as a real friend, was cheating everyone, including him. Accordingly, the direct address is employed to modify the text's narrative and not, as commonly used, connect diegetic and extra-diegetic dimensions.

Moreover, the analysis findings have also brought to light that when the direct address is used creatively, as in the second example, it gives rise to surprising effects on intersubjectivity. The building and sharing of what we have called “intersubjective platform of intra-diegetic nature” and “intersubjective intra-diegetic platform” is a clear example of what such a dramaturgical technique can bring about to develop the story and fill up the divide with the audience when strategically used.

The polydimensional analytical approach used in this study has shown that the direct address technique gives birth to what we could call a “mediated conversation,” a specific place where diegetic and extra-diegetic dimensions in TV series are articulated. The integration of the pragma-dialectical approach to argumentation with the interlocutory logic theory allowed us to see from different angles the intertwine of argumentative strategies and illocutionary characterization. The link between argumentative analysis and illocutionary analysis is here considered from the point of view of the role played by preparatory conditions and sincerity conditions on the production and organization of arguments in direct address. The analysis of the illocutionary layer of the two examples has shown the importance of these two family of conditions. The point of articulation between argumentative analysis and illocutionary analysis is constituted by the arguments, which constitute the preparatory conditions for Frank and Claire Underwood's speech acts. In our opinion, if we do not take into consideration these two elements brought to light in the analysis of the illocutionary layer, we cannot understand why Frank Underwood, in the first example, and Claire Underwood, in the second example, advance the arguments that we have reconstructed through argumentative analysis.

## Conclusions

In conclusion, the present study shows how an integrated approach based on argumentative and conversational tools of analysis permits to enlarge the traditional media studies perspective, highlighting the less known polydimensional structure and analyzing the psychosocial functions of the direct address, here considered as a research object of the social psychology of communication examined in its diegetic and extra-diegetic dimensions. The integration of argumentation theory and illocutionary analysis is a clear gain for researchers who are interested in studying media also from an “outer” perspective, not bounding themselves to the “media text,” but opening themselves to the “media context,” that is the social scene on which nowadays—via the social media—an essential part of the fortune of tv series is determined. Through the integration of the pragma-dialectical approach to argumentation (van Eemeren and Grootendorst, 1992; van Eemeren, 2010) with the interlocutory logic theory (Trognon and Batt, 2010), we have shown that the two direct addresses analyzed open and close the circle between “inside” and “outside” the narration, establishing a new modality of use of the direct address that, in our opinion, can be termed “intersubjective aside,” a type of aside that can be added to the three known and already described at the beginning of this work, i.e., aside *ad spectatores*, monological aside, and dialogical aside.

This study constitutes the first step toward a further articulation of the argumentative and conversational tools of analysis. Their improved integration will allow us to work on a wide range of research problems traditionally studied in many different scientific fields. This endeavor would have a 2-fold objective: at the level of data production, it is to improve the definition of the research objects to be investigated; at the level of data analysis, it is to adopt analytical tools able to increase our understanding of the complexity of our research objects from an integrated argumentative and conversational perspective.

## Data Availability Statement

The original contributions presented in the study are included in the article/supplementary material, further inquiries can be directed to the corresponding author/s.

## Author Contributions

All authors listed have made a substantial, direct and intellectual contribution to the work, and approved it for publication.

## Conflict of Interest

The authors declare that the research was conducted in the absence of any commercial or financial relationships that could be construed as a potential conflict of interest.

## Appendix

### Transcriptions Conventions

**-** cut off of the prior word or sound
**( )** description of situation/speaker's actions
**word** forms of stressing (pitch and/or volume)
**(0.1)** elapsed time in tenths of seconds
= lack of interval between the end of a prior and start of a next piece of talk
**(h)** explosive aspiration
**°( )** low in volume
**:** prolonging of sounds
**// //** segments overlapped by the talk of another

## References

[B1] Beversluis J. (1971). “I Know”: an illocutionary analysis. Southern J. Philos. 9, 345–351. 10.1111/j.2041-6962.1971.tb02147.x

[B2] Birke D. Warhol R. (2017). Multimodal you: playing with direct address in contemporary narrative television, in How to Do Things with Narrative: Cognitive and Diachronic Perspectives, eds Alber J. Olson G. (Berlin, Boston, MA: De Gruyter), 141–156. 10.1515/9783110569957-010

[B3] Bova A. (2019). Dialogical construction of parental feeding strategies during family mealtimes. J. Health Psychol. 10.1177/1359105319884600 31665938

[B4] Brown T. (2012). Breaking the Fourth Wall: Direct Address in the Cinema. Edinburgh: Edinburgh University Press.

[B5] Condoravdi C. Lauer S. (2012). Imperatives: meaning and illocutionary force. Empirical Issues Syntax Seman. 9, 37–58.

[B6] Galimberti C. Spanò C. (2017). Intersubjectivity in media consumption as a result of the relation between texts and contexts: The cases of Game of Thrones. Essais 12, 191–208. 10.4000/essais.3091

[B7] Gerbaz A. (2008). Direct Address, ethical imagination, and errol morris's interrotron. Film Philos. 12, 17–29. 10.3366/film.2008.0013

[B8] Hilmes M. (1985). The television apparatus: direct address. Jo. Film Video 37, 27–36.

[B9] Kinney K. (2019). Facing the camera: black actors and direct address in independent films of the 1960s. J. Cine. Media Stud. 59, 66–88. 10.1353/cj.2019.0063

[B10] Klarer M. (2014). Putting television ‘aside’: novel narration in house of cards. New Rev. Film Televis. Stud. 12, 203–220. 10.1080/17400309.2014.885818

[B11] Kuhn D. (1991). The Skills of Argument. New York, NY: Cambridge University Press. 10.1017/CBO9780511571350

[B12] Marriott S. (2007). Live Television: Time, Space and the Broadcast Event. London: Sage Publications.

[B13] Mittell J. (2006). Narrative complexity in contemporary american television. Velvet Light Trap 58, 29–40. 10.1353/vlt.2006.0032

[B14] Pfister M. (1991). The Theory and Analysis of Drama. Cambridge: Cambridge University Press.

[B15] Rigotti E. Greco Morasso S. (2009). Argumentation as an object of interest and as a social and cultural resource, in Argumentation and Education: Theoretical Foundations and Practices, eds Muller Mirza N. Perret-Clermont A. N. (New York, NY: Springer), 9–66. 10.1007/978-0-387-98125-3_2

[B16] Sacks H. Schegloff E. A. Jefferson G. (1974). A simplest systematics for the organisation of turn-taking for conversation. Language 50, 696–735. 10.1353/lan.1974.0010

[B17] Searle J. Vanderveken D. (1985). Foundations of Illocutionary Logic. Cambridge: Cambridge University Press. 10.1007/1-4020-3167-X_5

[B18] Tindale C. W. (2015). The Philosophy of Argument and Audience Reception. Cambridge: Cambridge University Press. 10.1017/CBO9781316181645

[B19] Trognon A. (2002). Speech acts and the logic of mutual understanding, in Essays in Speech Acts Theory, eds Vanderveken D. Kubo S. (Amsterdam: John Benjamins and Sons), 121–133. 10.1075/pbns.77.08tro

[B20] Trognon A. Batt M. (2010). Interlocutory logic. A unified framework for studying conversational interaction, in New Adventures in Language and Interaction, ed Streeck J. (Amsterdam: John Benjamins), 9–46. 10.1075/pbns.196.02tro

[B21] van Eemeren F. H. (2010). Strategic Maneuvering in Argumentative Discourse. Amsterdam, Philadelphia, PA: John Benjamins 10.1075/aic.2

[B22] van Eemeren F. H. Grootendorst R. (1992). Argumentation, Communication, and Fallacies. A Pragma-Dialectical Perspective. Hillsdale, NJ: Erlbaum.

[B23] van Eemeren F. H. Grootendorst R. (2004). A Systematic Theory of Argumentation: The Pragma-dialectical Approach. Cambridge: Cambridge University Press. 10.1017/CBO9780511616389

[B24] van Eemeren F. H. Houtlosser P. (2002). Strategic manoeuvring: Maintaining a delicate balance, in Dialectic and Rhetoric: The Warp and Woof of Argumentation Analysis, eds van Eemeren F. H. Houtlosser P. (Dordrecht: Kluwer), 131–159. 10.1007/978-94-015-9948-1_10

[B25] Vanderveken D. (1990). Meaning and Speech Acts: Volume 1, Principles of Language Use. New York, NY: Cambridge University Press.

[B26] Walton K. L. (1990). Mimesis as Make-believe: On the Foundations of the Representational Arts. Cambridge, London: Harvard University Press.

[B27] Weigand E. (2006). Argumentation: the mixed game. Argumentation 20, 59–87. 10.1007/s10503-006-9000-4

[B28] Woods F. (2019). Too close for comfort: direct address and the affective pull of the confessional comic woman in chewing gum and fleabag. Communic. Cult. Crit. 12, 194–212. 10.1093/ccc/tcz014

